# Clinical Presentation of Postnatally Acquired Cytomegalovirus Infection in Preterm Infants—A Case Series Report

**DOI:** 10.3390/children12070900

**Published:** 2025-07-08

**Authors:** Dobrochna Wojciechowska, Dominika Galli, Justyna Kowalczewska, Tomasz Szczapa, Katarzyna Ewa Wróblewska-Seniuk

**Affiliations:** 1 II Department of Neonatology, Poznan University of Medical Sciences, 61-701 Poznań, Poland; tszczapa@ump.edu.pl (T.S.); kwroblewska@ump.edu.pl (K.E.W.-S.); 2 Doctoral School, Poznan University of Medical Sciences, 61-701 Poznań, Poland; 3 Student Scientific Society of Neonatology and Perinatology, Poznan University of Medical Sciences, 61-701 Poznań, Poland; 83681@student.ump.edu.pl (D.G.);

**Keywords:** newborn, preterm, acquired cytomegalovirus infection, postnatal infection, low birth weight, breast milk, neonatal intensive care, cytomegalovirus, case report

## Abstract

**Background**: Human cytomegalovirus (HCMV) is the leading cause of congenital and acquired viral infections in newborns. While acquired infections are often asymptomatic, premature infants—especially those born before 30 weeks of gestation or with a very low birth weight (<1500 g)—are at an increased risk for severe infections. These can manifest as thrombocytopenia, liver failure, sepsis-like symptoms, and, in rare cases, death. HCMV is transmitted through various human secretions, including breast milk, which is the optimal feeding method for premature infants. **Methods:** We present five premature neonates, born between 23 and 26 weeks of gestation, each with a distinct clinical presentation of acquired HCMV infection. **Results:** All infants tested negative for congenital CMV infection via molecular urine testing within the first three weeks of life. Acquired infection was diagnosed between the second and third month of life, with symptoms such as septic shock, persistent thrombocytopenia, and signs of liver failure. Each infant received antiviral treatment along with regular viral load monitoring. Unfortunately, one patient died due to complications of prematurity. The remaining infants were discharged and continue to receive follow-up care in an outpatient clinic. **Conclusions:** These cases of postnatally acquired CMV infection aim to increase awareness of its highly heterogeneous and nonspecific clinical presentation, which may result in an incorrect, delayed, or concealed diagnosis. Currently, there are no clear guidelines on how to manage the presence of the virus in maternal breast milk, particularly for premature infants. It should be recommended to perform a molecular CMV test in all breast-fed preterm infants who present with sepsis-like symptoms, thrombocytopenia, liver failure, or other organ involvement. In case of a confirmed aCMV diagnosis, appropriate treatment should be introduced.

## 1. Introduction

Human cytomegalovirus is the most common congenital infection. However, postnatal mother-to-child transmission, primarily through breastfeeding, appears to have even greater epidemiological significance [1]. Although postnatally acquired cytomegalovirus (aCMV) infection is usually not clinically significant in full-term newborns, very premature infants, particularly those born before 30 weeks of gestation or with a very low birth weight (<1500 g), are vulnerable to severe outcomes. These infants may develop cholestasis, pneumonia, bowel inflammation, severe thrombocytopenia, neutropenia, and symptoms resembling generalized infection (sepsis-like syndrome), and in rare cases, the infection can be fatal [2,3].

HCMV is widespread in the human population and can be transmitted through various body fluids, including blood, saliva, cervical secretions, and breast milk. While the exact transmission mechanism between mother and infant is not fully understood, recent studies suggest that viral reactivation may occur in the mammary gland. Notably, the mother may show no symptoms or evidence of systemic reactivation. In seropositive women, the transmission rate of HCMV to the infant via breast milk is approximately 40% [3].

Despite this, there are no clear guidelines on how to manage the presence of HCMV in maternal breast milk, which remains the preferred feeding method, especially for preterm infants, due to its numerous nutritional benefits [4].

This study aimed to present several cases of extremely premature neonates with varying clinical manifestations of aCMV infection.

## 2. Case Reports

This retrospective study was conducted in a single tertiary neonatal care unit at the Gynecological-Obstetrical University Hospital in Poznan, Poland. The inclusion criteria were a birth gestational age of <28 weeks and a diagnosis of acquired cytomegalovirus infection. Infants diagnosed with congenital CMV infection were excluded from the study. PCR CMV urine testing within the first three weeks of life, conducted to diagnose congenital infection, is the standard of care in our unit for all newborns born before 32 weeks of gestation.

aCMV infection was diagnosed between the 4th and 12th week of life based on a urine PCR test, blood test, and, in some cases, cerebrospinal fluid (CSF) test. The test toward CMV was performed as part of the differential diagnosis due to symptoms such as persistent thrombocytopenia, signs of liver failure, cholestasis, septic shock (with excluded bacterial etiology), and pneumonia. All patients were initially treated with intravenous ganciclovir, and the treatment was continued with oral valganciclovir, depending on the patient’s clinical condition. Regular viral load monitoring was performed, and the completion of treatment was determined by obtaining a negative result in the blood test.

Table 1 summarizes the key clinical data and the course of treatment of the patients described in this manuscript.

### 2.1. Patient 1

A female infant born at 25 weeks of gestation, with a birth weight of 615 g, via caesarean section due to placenta previa and placental abruption, in poor condition, had Apgar scores of 3, 6, and 6 at the 1st, 3rd, and 5th minute of life, respectively. The patient required intubation and mechanical ventilation immediately after birth and received surfactant via an endotracheal tube. For several days, she also required high-frequency oscillatory ventilation and inhaled nitric oxide (iNO) therapy.

A transfontanelle ultrasound revealed a grade I intraventricular hemorrhage and subsequent cerebellar hemorrhage.

Initially, total parenteral nutrition was started, and trophic feeding with maternal milk was introduced on the 3rd day. Enteral feeding was gradually increased, and parenteral nutrition was finished on the 92nd DOL.

At 3 months of age, the infant developed septic shock, with thrombocytopenia and elevated inflammatory markers (C-reactive protein (CRP) 148 mg/L, procalcitonin (PCT) >100 ng/mL, interleukin-6 (IL-6) 20,000 pg/mL). Broad-spectrum antibiotic therapy was introduced; however, blood and endotracheal aspirate cultures remained sterile. Molecular testing confirmed aCMV infection (2.17 × 10^5^ copies DNA/mL in blood). CMV was also detected in the mother’s breast milk (3.5 × 10^4^ copies DNA/mL). According to the medical history, the mother was CMV IgG-seropositive and IgM-seronegative during pregnancy.

Ganciclovir therapy was initiated and continued for 3 weeks, resulting in a gradual decrease in viral load. During CMV treatment, maternal breast milk feeding was changed to formula. After completing the therapy, when proven CMV-negative, the patient was fed with pasteurized and frozen breast milk.

After 151 days of hospitalization, the infant was discharged home.

Currently, she is twelve months old (corrected age nine months), remains under specialist care (neurologist, neonatologist, pulmonologist), and continues medical rehabilitation.

### 2.2. Patient 2

A male newborn, born at 23 weeks of gestation, with a birth weight of 620 g, was delivered vaginally, in poor general condition, with Apgar scores of 2, 4, 6, and 8 at the 1st, 3rd, 5th, and 10th minute of life. Immediately after birth, the newborn was intubated, and mechanical ventilation was started; he received two doses of surfactant. Due to the symptoms of persistent pulmonary hypertension, iNO therapy was initiated, along with high-frequency oscillatory ventilation. Given the mother’s medical history, empirical antibiotic therapy was started immediately after birth. On the 2nd DOL, a chest X-ray showed lung parenchymal bleeding.

Initially, the newborn received total parenteral nutrition and trophic feeding with maternal milk. Enteral feeding with the mother’s breast milk and milk from a human milk bank was gradually increased starting from the 8th DOL.

At 2 months of age, persistent thrombocytopenia and signs of liver failure prompted investigation, and PCR testing confirmed CMV viremia (5.4 × 10^5^ copies DNA/mL). CMV DNA was also detected in the mother’s breast milk (3.1 × 10^4^ copies DNA/mL), indicating CMV reactivation, as the mother was CMV IgG-seropositive and IgM-seronegative before pregnancy.

Ganciclovir therapy was initiated and continued for 45 days until the viremia was resolved, with dosage adjustments due to severe leukopenia.

Given the CMV infection and the virus presence in maternal milk, the infant’s diet was changed to a combination of milk from the human milk bank and hydrolyzed, hypoallergenic infant formula. After completing CMV treatment, feeding continued using formula. A transfontanelle ultrasound revealed extreme brain immaturity and grade II intraventricular hemorrhage. An electroencephalogram (EEG) revealed seizure activity, prompting the initiation of antiepileptic treatment. Brain magnetic resonance imaging (MRI) confirmed severe hypoxic–ischemic changes. Despite aggressive treatment, the infant’s condition continued to deteriorate, with progressive circulatory and respiratory decompensation, renal failure, massive hypoxic brain injury, and significant metabolic and electrolyte imbalances.

On the 150th day of life, after several discussions within the medical team and with the parents, the decision was made to transition to palliative care. The infant passed away on the 153rd DOL.

### 2.3. Patient 3

A male newborn, born vaginally at 25 weeks of gestation, with a birth weight of 850 g, presented with poor condition, with Apgar scores of 2, 2, 6, and 7 at the 1st, 3rd, 5th, and 10th minute of life. He was intubated and mechanically ventilated and received one dose of surfactant. On the 2nd DOL, despite initial empiric broad-spectrum antibiotic therapy, he developed a generalized infection of ESBL-producing *Klebsiella pneumoniae* etiology. Transcranial ultrasound showed intraventricular bleeding, with progression to grade IV. On the 33rd DOL, a Rickham reservoir was inserted without complications. The neurological state of the infant was abnormal, with hypotonia, seizures, and tremors.

Feeding was started with partial parenteral nutrition and gradually increasing portions of the mother’s breast milk.

On the 41st DOL, the infant’s condition deteriorated, with fever, elevated inflammation markers (CRP 93 mg/L, PCT 0.81 ng/mL), and asymptomatic thrombocytopenia (83.0 G/L). Broad-spectrum antibiotics and antifungals were administered; however, blood and cerebrospinal fluid cultures were negative. The CMV PCR urine test was positive. The virus was also detected in CSF (4.39 × 10^4^ copies DNA/mL) and blood (5.44 × 10^5^ copies DNA/mL) as well. The mother’s breast milk also tested positive for CMV. It was known that the mother was CMV IgG-seropositive and IgM-seronegative during pregnancy. Intravenous treatment using ganciclovir was carried out for 39 days and later continued orally with valganciclovir during hospitalization and after discharge. During CMV infection, the infant was fed partially parenterally due to the severe general condition and poor toleration of oral feeding with formula and then pasteurized and frozen mother’s breast milk

In the third month of life, we noted a severe exacerbation of retinopathy of prematurity (ROP); aggressive posterior retinopathy of prematurity (AP-ROP) was diagnosed. The patient was treated with anti-VEGF monoclonal antibody (Lucentis), followed by retinal laser photocoagulation at 4 months of age due to inadequate response.

The patient was discharged home on the 109th DOL in good general condition.

Valganciclovir treatment was continued under the supervision of an infectious disease specialist for another 6 months, until a negative blood viral load result was obtained at 8 months of age. At 7 months, the Rickham reservoir was replaced with a ventriculoperitoneal valve. Currently, he is twelve months old (corrected age nine months). He remains under specialist care (neurologist, neonatologist, ophthalmologist) and continues medical rehabilitation.

### 2.4. Patient 4

A female newborn, born at 26 weeks of gestation, with a birth weight of 800 g, via caesarean section due to fetal distress, received Apgar scores of 6, 7, and 7 at the 1st, 3rd, and 5th minute of life. The infant was intubated in the delivery room, and mechanical ventilation was started. She received two doses of surfactant; inhaled nitric oxide was administered for pulmonary hypertension.

Given the maternal history of genital tract infections, broad-spectrum antibiotics were started and continued for 7 days. The infant was initially fed parenterally; trophic feeding with the mother’s milk was introduced on the 2nd DOL, and feeding volumes were gradually increased. In the fourth week of life, ventilator-associated pneumonia (VAP) caused by *Enterobacter hormaechei* and *Acinetobacter* species was diagnosed, and targeted antibiotic therapy was initiated. However, the inflammatory markers remained relatively low—PCT 1.79 ng/mL, CRP 2.1 mg/L.

At the same time, the mother’s milk was revealed to be PCR positive for CMV, and an antibody assay in the mother showed reactivity in both antibody classes (IgG 143.0 U/mL, IgM 1.22 U/mL). On the 26th DOL, the infant’s urine and blood tested positive for CMV, which confirmed aCMV infection. Intravenous ganciclovir was started, switched to oral valganciclovir after 6 days and continued for 2 weeks, until the CMV viral load became undetectable. During CMV treatment, a gradual improvement in respiratory function was observed. The infant was extubated on the 34th DOL and continued noninvasive ventilation, achieving respiratory stability by the 110th day. A slow resolution of inflammatory changes was observed via chest X-ray and chest ultrasound.

On the 115th DOL, the infant was discharged home in good general condition. At seven months of age (corrected age four months), she remains under specialist care (neurologist, neonatologist) and continues medical rehabilitation.

### 2.5. Patient 5

A male newborn, born at 26 weeks of gestation, with a birth weight of 800 g, via caesarean section due to incipient HELLP syndrome in the mother, in serious general condition, received Apgar scores of 1, 3, 3, and 5 at the 1st, 3rd, 5th, and 10th minute of life. He was intubated in the delivery room, and mechanical ventilation was started; one dose of surfactant was administered.

Due to the infant’s critical condition and risk factors for intrauterine infection, empirical broad-spectrum antibiotic therapy was started. On the 3rd DOL, we observed massive pulmonary bleeding; a second dose of surfactant was given, and the ventilation mode was switched to high-frequency oscillatory ventilation for several days.

On the 7th DOL, gastrointestinal perforation occurred. It was treated with peritoneal cavity drainage and second-line antibiotics, given the infant’s severe condition. Trophic feeding was started on the 22nd DOL, and by the 46th DOL, the infant was fed exclusively enterally.

Persistent cholestasis prompted testing for CMV infection. At the 9th week of life, PCR CMV testing of the infant’s urine and blood was positive (1.2 × 10^5^ copies DNA/mL), confirming an aCMV infection. CMV was also detected in maternal milk (1.82 × 10^3^ copies DNA/mL). The mother had never been serologically tested for CMV infection before. After that, the baby was fed with milk from a human milk bank and later with maternal milk, frozen and pasteurized to mitigate the risk of viral transmission.

The infant was treated with oral ganciclovir for 26 days, and the viral load gradually became undetectable by the end of the treatment.

A transfontanelle ultrasound revealed a grade III intraventricular hemorrhage with periventricular infarction on the left side and features of periventricular leukomalacia (PVL).

Retinopathy of prematurity (ROP grade 3) was diagnosed, and retinal laser therapy was performed.

On the 95th DOL, the infant was discharged home in good general condition.

At seven months of age (corrected age four months), he remains under specialist care (neurologist, neonatologist, ophthalmologist) and continues medical rehabilitation.

## 3. Discussion

In this case report study, we described five extremely premature infants diagnosed with aCMV during their stay in the neonatal intensive care unit. Each of our patients presented a different clinical picture of the disease. We described the signs and symptoms of the disease, the diagnostic path that allowed us to diagnose the disease, the treatment used, and the infection outcome.

Cytomegalovirus is a leading cause of congenital infections, and the postnatal acquisition of CMV also poses a significant concern, particularly for very low birth weight and preterm infants. A growing body of research attempts to understand the risk factors, transmission mechanisms, and clinical outcomes of CMV infection in this vulnerable population.

### 3.1. Importance of Early CMV Screening in Preterm Infants: Differentiating Congenital from Acquired Infections

CMV infections in neonates can be either congenital or acquired. Congenital CMV refers to the infection that a neonate is born with, whereas acquired CMV occurs postnatally, most often due to exposure to the virus after birth. Early screening for cytomegalovirus in the first days of life in preterm infants is crucial for several reasons. Preterm infants, particularly those born at very low birth weight (VLBW), are at a heightened risk of acquiring CMV during their hospital stay, mainly due to feeding via breast milk from CMV-seropositive mothers [5]. The distinction between congenital and acquired CMV infection is critical for making informed decisions about treatment, management, and long-term prognosis. cCMV infection can be detected through urine or saliva samples obtained within the first two to three weeks of life [6,7]. This early screening is essential for differentiating between congenital and acquired infections in preterm infants. If CMV is detected in the neonate’s urine via PCR testing, it indicates a congenital infection, and further diagnostic tests can be used to assess the potential involvement of other organs or systems (e.g., the brain, liver, eyes, or ears). Congenital CMV is associated with more severe long-term neurodevelopmental outcomes, such as hearing loss, cognitive delays, and visual impairment [7]. aCMV infection, on the other hand, typically occurs after birth and can be diagnosed through repeated PCR testing of the infant’s urine or blood once the infant reaches a few weeks of age, mainly if clinical signs of infection (such as sepsis-like syndrome, pneumonia, or thrombocytopenia) are observed [8]. Differentiating between congenital and acquired CMV is important because the clinical presentation and potential complications can vary significantly between the two types of infection.

### 3.2. CMV Transmission via Human Milk: Prevalence and Risk Factors

Acquired cytomegalovirus (aCMV) infection can be transmitted through several routes, most notably via breast milk, blood transfusions, and direct contact with bodily fluids such as saliva and urine. Among preterm infants, postnatal transmission through breast milk from seropositive mothers is the most common route, with viral shedding detected in up to 97% of lactating women and infection rates in very low birth weight infants ranging from 11% to 40% [9,10]. Transmission via blood products has been significantly reduced through CMV-seronegative donor selection and leukoreduction techniques [11]. In older children and adults, CMV spreads through close contact with infected secretions and sexual transmission [12]. Although rare, nosocomial transmission in healthcare settings remains a theoretical concern due to potential exposure to infectious fluids [13].

In the patients described in this manuscript, CMV DNA was measured in breast milk samples, with viral presence confirmed in all cases, suggesting breast milk as a likely postnatal source of infection. In our hospital, all blood products administered to the infants are leukoreduced and CMV-seronegative, which effectively minimizes the risk of transfusion-transmitted CMV. Nosocomial transmission was considered unlikely due to strict infection control protocols in place and the absence of other concurrent CMV cases within the neonatal unit during the study period.

Breast milk is advised as the only source of nutrition for the first six months of life and is considered the gold standard for newborn feeding, beginning in the first few hours of life. It contains macro- and micronutrients and bioactive substances such as immunoglobulins, cytokines, growth factors, and hormones. The immunomodulatory function of breast milk lowers the risk of infection, strengthens the intestinal barrier, and protects against ROP, BPD (bronchopulmonary dysplasia), and NEC (necrotizing enterocolitis).

While human milk is an essential source of nutrition for preterm infants, the transmission of CMV through breast milk remains a key challenge. In CMV-seropositive women, the virus may reactivate locally in the breasts during lactation and can be excreted in the milk. It can be detected even in colostrum [14]; however, in most women, CMV reactivation starts about 10 days after delivery. The initial positive result of CMV DNA by PCR in the mother’s breast milk is typically found within the first two weeks of lactation [15]. Between 4 and 6 weeks postpartum, cytomegalovirus can be isolated from breast milk in up to 100% of HCMV IgG-positive mothers [16], while by the 8th week, it typically becomes undetectable [15]. The transmission rate of CMV through breast milk is 58–69% in term infants, 5.7–58.6% in preterm infants, and 38% in preterm infants with a birth weight less than 1500 g or gestational age under 32 weeks. The risk of aCMV infection may be higher in populations with a high prevalence of seropositivity [17].

All patients in the study received unpasteurized breast milk from their mothers starting on the first days of life. We assume that it was the source of their infection since the virus was also detected in all mothers’ breast milk. Other authors of aCMV case reports also underline this route of infection [18].

Freezing and pasteurizing milk is a crucial intervention to reduce transmission and mitigate the risks associated with CMV infection. A meta-analysis of 17 studies by Lanzieri et al. found that freezing breast milk slightly lowered the CMV infection rate but did not significantly reduce the occurrence of CMV-related sepsis-like syndrome (CMV-SLS), indicating that freezing alone is not highly effective [9]. The Holder pasteurization method, which involves heating at 63–65 °C for 30 min, is the most effective for inactivating the virus; however, it diminishes the biological activity of several breast milk components, such as growth factors, immunoglobulins, and enzymes. Short-term high-temperature sterilization (72 °C for 5 s) also reduces CMV DNA and better preserves these beneficial components. While pasteurization has been shown to be effective in reducing the viral load of CMV in milk, the study concluded that more research is needed to determine the most effective duration and temperature of the process to enable simultaneous reduction in the viral load and preservation of breast milk’s beneficial properties [5].

This corroborates the findings from Hu et al., who also demonstrated that untreated milk carries a significantly higher risk of CMV infection compared to frozen or pasteurized milk [19].

The balance between the benefits of exclusive breast milk feeding, such as improved immune protection and better neurodevelopment, and the risks of CMV transmission remains a critical area for further investigation [5].

The current recommendations of the American Academy of Pediatrics state that the evidence is insufficient to support withholding a mother’s own milk (MOM) in very low birth weight VLBW infants because of the risk of aCMV [20].

In Germany, it is recommended that VLBW infants receive sterile breast milk for the first 6 weeks of life. The Austrian Academy of Pediatrics advises that CMV-seropositive mothers continue sterilizing their milk until 34 weeks postconception. In France, sterilization is recommended until 32 weeks of corrected age for preterm infants born before 28 weeks of gestation and weighing under 1000 g, if the mother’s CMV antibody status is positive or unknown; otherwise, fresh breast milk is recommended. On the other hand, some suggest that freezing alone may be adequate, as CMV infection via breast milk is rare and long-term complications are not a major concern [5]. In addition, the ESPGHAN Committee in 2022 issued the following position statement: “There is insufficient evidence to determine whether potential sequela of aCMV transmission is more harmful than potential adverse effects arising from providing pasteurized mother’s own milk instead of fresh MOM. Thus, while we acknowledge the potential adverse consequences of aCMV, especially in the most immature infants, we do not recommend routinely pasteurizing MOM from CMV-positive women as this may reduce the beneficial effects of fresh MOM” [21].

In the group of patients we described, all babies initially received both fresh and frozen breast milk. Freezing at −18 to −20 °C of the mother’s breast milk was used if there was an oversupply, then thawed milk was given to the newborn if the mother could not provide a fresh portion. According to the current recommendations of the Polish Society of Neonatology, it is not recommended to routinely freeze or pasteurize the breast milk of CMV IgG-positive mothers [22]. However, once the infection was diagnosed in our patients, and the viral load was confirmed in the mother’s milk, the thermal processing procedure was applied according to the local human milk bank protocol. It included pasteurization at 62.5 degrees Celsius for 30 min and then gradual cooling to 4 degrees Celsius and freezing at −18 to −20 degrees Celsius. Such a procedure was implemented only after the diagnosis of infection in the presented patients and continued until the viral load in breast milk was negative.

### 3.3. CMV Diagnosis: Screening Challenges and Sensitivity of Testing

Accurate and timely diagnosis of postnatal CMV infection also remains a challenge. Many studies have explored various diagnostic tools, including the use of maternal milk, infant saliva, and urine samples for detecting CMV. The study by Mukhopadhyay et al. highlights the potential of saliva and maternal breast milk to serve as diagnostic tools for identifying maternal serostatus and detecting aCMV infection in infants [23]. While maternal milk was found to be highly sensitive in identifying CMV serostatus, saliva samples showed only limited sensitivity (30%) in detecting CMV infection in preterm infants. This is likely due to the lower viral load in saliva than urine and the difficulty associated with collecting an adequate saliva volume for testing (especially in VLBW infants). Moreover, it is worth bearing in mind the risk of receiving a false positive test result due to the possible contamination of the baby’s saliva with the mother’s breast milk, where copies of the virus are actively secreted [23].

In our department, routine diagnostic tests for cCMV infection are performed for all premature babies born under 32 weeks of gestation: within the first 3 weeks of life, a urine sample is collected, and a PCR test is performed to rule out congenital infection. A urine sample is collected again if a suspicion of acquired infection is raised. Blood and cerebrospinal fluid are tested if the urine tests are positive for CMV. In order to monitor the severity of the infection and treatment effectiveness, quantitative CMV DNA tests are performed at this point in the diagnostic sequence. Such a strategy is recommended by the Polish Neonatal Society [22]. Even though congenital cytomegalovirus infection is relatively rare in this group of patients, such a test allows to distinguish between congenital and acquired CMV if aCMV is suspected at a later time.

It is not common practice in our unit to test the mother’s breast milk for the presence of CMV DNA. However, since we have observed more cases of aCMV in the last years, such a standard of care should be implemented. In other units, apart from testing premature infants born before 32 weeks of gestation for cCMV by means of a PCR CMV urine test, they also test the breast milk from their mothers, between the 10th and 14th day after birth, to detect CMV reactivation [1].

Another solution would be to feed premature infants with pasteurized milk. There are units where preterm infants from the 8th day of life to a negative breast milk CMV PCR result (or up to 34 PMA) receive pasteurized milk [1]. However, it is known that such a procedure diminishes the biological activity of several milk components, so it seems not to be an optimal choice.

### 3.4. Clinical Impact: Morbidity and Mortality

The clinical impact of postnatal CMV infection is multifaceted. While in some infants, infection remains asymptomatic, in others, it manifests severely with a wide range of clinical symptoms like NEC, BPD, or accelerated development of retinopathy of prematurity ROP [24,25].

In two of our patients, aCMV was diagnosed when infants presented with sepsis-like syndrome associated with thrombocytopenia and elevated inflammatory markers. Since the bacterial cultures were negative, we searched for other potential causes of the exacerbation. A similar case was reported by Takahashi et al. [26], who highlighted that the infant had been exclusively fed breast milk previously frozen at –20 °C prior to administration. This finding suggests that freeze–thawing may not reliably inactivate cytomegalovirus (CMV), particularly in the context of high viral loads, and may therefore be insufficient as a sole preventive strategy in at-risk neonatal populations [26]. In the patient described by Fischer et al., sepsis-like syndrome was associated with multiorgan involvement in the form of pneumonitis and enterocolitis. In one of our patients, we also observed pneumonia, which was initially thought to be of bacterial etiology. However, it did not improve with target antibiotic therapy and only when ganciclovir treatment was finally introduced. Even though we did not observe colitis in any of our patients, in two of them, we noted poor enteral feeding tolerance at the time of aCMV infection, which improved with antiviral treatment.

Other symptoms that might prompt the diagnostics toward aCMV are cholestasis and signs of liver failure, especially if accompanied by thrombocytopenia. Such a clinical picture was noted in two patients of our set and is also presented by other authors.

It has been shown that infants diagnosed with aCMV are at increased risk of retinopathy of prematurity due to the release of cytokines stimulating the production of growth factors that can intensify vascular proliferation and ROP progression. In their study, Maria Josa et al. suggest the need for diagnostic testing for aCMV in infants with ROP exacerbation. Based on the case series, they also suggest that the inclusion of antiviral treatment reduces the risk of severe ROP, which requires surgical treatment [27].

We observed a significant worsening of the course of ROP, with progression to AP-ROP during aCMV infection in one of the patients. The child required anti-VEGF factor (Lucentis) injections into both eyes and then bilateral retinal laser photocoagulation due to the gradual progression of ROP. In another patient, we also observed an exacerbation of retinopathy during cytomegalovirus infection. Shortly after CMV infection diagnosis, grade 3 ROP was observed, and the patient required retinal laser photocoagulation for both eyes. In contrast to the Josa team’s reports cited above, we have not observed any improvement in retinopathy onset after introducing antiviral treatment. It is, however, difficult to confirm that ROP was due to CMV infection, given other risk factors of retinopathy observed in the patient, such as low gestational age and low birth weight, as well as long-term supplemental oxygen therapy.

There are also case reports that show the coincidence of aCMV and retinitis. Tajalli et al. present a case of a premature female infant where, upon screening for retinopathy of prematurity, diffuse occlusive vasculitis was detected, and at the same time, a CMV was confirmed [25]. Piersigilli presented another case of severe retinitis with retinal hemorrhages and retinal detachment [28]. In both cases, antiviral therapy was started, and improvement was observed. Even though ocular changes are typical for congenital CMV, in infants with aCMV, both retinitis and ROP exacerbation are also observed.

BPD is a prevalent and serious medical condition commonly linked to preterm birth. Besides impaired proximal airway and bronchoalveolar development, BPD is often associated with pulmonary vascular disease (PVD) [29]. The abnormalities in the developing pulmonary vasculature might not be severe enough to be clinically identified as pulmonary hypertension, yet they can lead to impaired gas exchange and changes in pulmonary blood flow distribution, particularly in response to acute respiratory infections [30].

Stanford et al. highlight the relationship between CMV and PVD in extremely premature infants, where CMV infection may exacerbate cardiorespiratory instability and worsen the trajectory of PVD, causing pneumonitis and derangement of pulmonary vascular development directly through endothelial dysfunction, particularly when it is not diagnosed and treated promptly [31]. Additionally, the systematic review by Guo et al. showed that CMV infection was significantly and positively associated with BPD diagnosed at 36 weeks of postmenstrual age [32]. In the group of patients we collected, we observed a severe course of bronchopulmonary dysplasia in three patients. However, it should be highlighted that all these infants also had many other risk factors for the development of BPD. The long-term effects of aCMV infection, particularly regarding neurodevelopmental outcomes, remain largely underexplored. In contrast to the indisputable effect of cCMV infection on the risk of abnormal neurological development (including hearing loss and cognitive delays), regardless of the trimester in which the infection occurred [8], the impact of aCMV on the risk of developmental abnormalities, especially in children born prematurely and with VLBW, is not clearly understood.

In the study of Pellkofer et al., the impact of aCMV infection on brain injury and microstructural brain maturation in very preterm infants at term-equivalent age has not been shown [33]. The research involved 401 infants born before 32 weeks of gestation, including 18 (4.5%) diagnosed with postnatal CMV infection. The study used MRI scans to compare brain injury and brain maturation markers (fractional anisotropy and apparent diffusion coefficient) in 14 brain regions between CMV-infected and noninfected infants. The results showed no significant differences in brain injury or brain development between the two groups, including infants born before 28 weeks of gestation [33].

### 3.5. Treatment Strategies

There is limited evidence supporting the effectiveness of antiviral treatment for children with aCMV infection. Due to the absence of clear links to long-term complications, treatment is generally reserved for cases of severe disease [34].

Treating premature infants with aCMV infection primarily aims to suppress viremia and prevent organ damage. The efficacy of antiviral treatment for aCMV infection remains an area of active research. Several case reports have described the use of ganciclovir antiviral therapy for aCMV with varying outcomes. However, no systematic studies have been conducted to determine the optimal dose, therapy duration, or overall treatment efficacy for aCMV. Ganciclovir (administered intravenously) and its prodrug, valganciclovir (administered orally), are considered the preferred antiviral treatment for CMV infections [24].

Recommended doses in neonates are 6 mg/kg every 12 h for ganciclovir and 16 mg/kg every 12 h for valganciclovir. Treatment typically lasts at least 2 weeks, with a reevaluation of symptoms and CMV viral load. Treatment may be extended up to 8 weeks, depending on the clinical condition of the patient and the laboratory test results [34]. Some infants may require longer therapy to resolve end-organ disease and CMV viremia. Consultation with an infectious disease specialist is recommended for these challenging situations [35]. In one of our patients, the therapy was prolonged up to 8 months of age until a negative blood viral load result was obtained. Other patients received shorter therapy, in agreement with the above-mentioned guidelines.

It is also important to remember the foreseeable side effects of treatment, such as bone marrow suppression leading to reversible neutropenia. Long-term toxicity is also a difficult aspect to study due to the difficulty of collecting data on the health of patients many years after the end of treatment [7].

Tajalli et al. [25] highlight that early diagnosis and prompt treatment with antiviral drugs may help mitigate the severity of CMV-related complications, such as CMV retinitis. However, challenges remain in ensuring timely detection and treatment in a population that is already at high risk of other neonatal morbidities [25].

In the group of patients we described, the choice of drug, ganciclovir vs. valganciclovir, depended on food tolerance and, consequently, the possibility of oral drug administration. In patient 2, a lower dose of ganciclovir, i.e., 3 mg/kg body weight, instead of the recommended dose of 6 mg/kg, was used due to observed neutropenia. The patient also temporarily required the administration of filgrastim (a form of recombinant human granulocyte colony-stimulating factor used to induce the production of granulocytes). Treatment with ganciclovir was continued until the peripheral blood was negative for viremia. In patient 3, the treatment was extended up to 6 months because of the persistent positive results of PCR CMV DNA tests. Such a decision was taken after consultation with the infectious disease specialist.

Moreover, there are no specific recommendations for the antiviral treatment of CMV-positive, non-HIV-infected breastfeeding mothers to prevent viral transmission via breast milk. Latent CMV DNA can persist in the host without causing active infection. However, intermittent viral shedding may occur throughout life, particularly during periods of immune suppression [36].

Concerning the efficacy of potential antiviral therapy, there is a study in HIV-positive, HSV-2 co-infected mothers. Valacyclovir treatment was safe but failed to reduce CMV viral load in breast milk or prevent infant postnatal CMV acquisition. The study suggests that stronger or alternative antiviral strategies may be needed to prevent CMV transmission via breastfeeding in this population [37].

Among the mothers of the patients described in this manuscript, none exhibited symptoms of infection during the period when viremia was detected in breast milk. Based on medical history, reactivation of the infection could be confirmed in all but one of the mothers, as seropositivity had been documented either prior to or at the onset of pregnancy. None of the mothers received antiviral treatment.

### 3.6. Long-Term Outcomes and Future Directions

According to Lawrence et al., historically, scientists thought that postnatal asymptomatic HCMV infection and latency presented minimal health risks to the host. However, growing evidence now points to adverse health effects, particularly with advancing age, in the development of cardiovascular disease, as HCMV DNA has been found in atherosclerotic plaques. Additionally, HCMV has been linked to immune dysfunction and frailty in aging populations [8].

Some studies have reported poor neurocognitive outcomes in adolescents born preterm with aCMV infection occurring during the hospital stay [17]. They may include reduced cognitive performance, particularly in complex cognitive functions [38]. The cognitive deficits observed, especially in visuomotor integration, may affect academic performance. These impairments appear to be additional to the effects of preterm birth itself. Moreover, male adolescents with CMV infection showed more pronounced cognitive deficits than females, suggesting possible sex differences in response to the infection [39].

Studies like Hu et al.’s suggest that we need more research on long-term sequelae, such as neurological and cognitive sequelae and sensorineural hearing loss, to clearly determine aCMV health risks [35]. Longitudinal studies tracking neurodevelopmental outcomes in CMV-infected preterm infants will be vital to developing evidence-based guidelines for the prevention and management of CMV infection in this population.

The strength of this study is that we collected a series of five extremely premature infants with different clinical pictures of the acquired cytomegalovirus infection. The analysis of these cases might be useful for other neonatologists and pediatricians in their everyday work.

## 4. Conclusions

The report of these several cases of postnatally acquired CMV infection aims to increase awareness of its highly heterogeneous and nonspecific clinical presentation, which may result in an incorrect, delayed, or concealed diagnosis. It needs to be highlighted that all breast-fed preterm infants of CMV-seropositive and of unknown status mothers who present with sepsis-like symptoms and such manifestations as thrombocytopenia, hepatosplenomegaly, cholestasis, pneumonia, or enterocolitis should have a molecular CMV PCR urine test performed and appropriate treatment introduced in case of the confirmed diagnosis. Postnatal CMV infection remains a significant concern for very low birth weight and premature infants, particularly in the context of breastfeeding. Pasteurization of breast milk has emerged as a key intervention to reduce the transmission of CMV, but uncertainties about its optimal implementation persist. Furthermore, while antiviral treatments may offer hope, the effectiveness and timing of such therapies require further exploration. Ultimately, a nuanced approach that balances the benefits of breastfeeding with the risks of CMV transmission will be essential in optimizing outcomes for VLBW and preterm infants. Future research should focus on refining diagnostic methods, improving prevention strategies, and understanding the long-term impacts of CMV on infant health and development.

## Figures and Tables

**Table 1:** Clinical Characteristics and Treatment Course of Patients Described in the Manuscript (CA—corrected age; DOL—day of life).

	Birth Weight (BW)	Gestational Age (GA)	CMV Viral Load (Urine/Blood/CSF)	CMV Viral Load (Breast Milk)	CMV Immunological Status of Mother	Treatment	Clinical Signs	Clinical Outcomes
**Patient 1**	615 g	25 2/7	64 DOL (urine): positive 65 DOL (blood): 2.17 × 10^5^ copies DNA/mL 80 DOL (blood): negative	67 DOL: 3.5 × 10^4^ copies DNA/mL 80 DOL: 5.85 × 10^4^ copies DNA/mL	IgG-seropositive and IgM-seronegative	Ganciclovir 6 mg/kg (21 days)	septic shock, with thrombocytopenia and elevated inflammatory markers (negative blood culture)	12 months (9 months CA), specialist care—neurologist, neonatologist, pulmonologist, medical rehabilitation
**Patient 2**	620 g	23 0/7	45 DOL (urine): positive 46 DOL (blood): 5.4 × 10^5^ copies DNA/mL 73 DOL (blood): 8.41 × 10^2^ copies DNA/mL 92 DOL (blood): negative	52 DOL: positive 81 DOL: 3.1 × 10^4^ copies DNA/mL 85 DOL: 3.51 × 10^4^ copies DNA/mL	IgG-seropositive and IgM-seronegative	Ganciclovir 3 mg/kg (45 days)	persistent thrombocytopenia and signs of liver failure	passed away on the 153rd DOL
**Patient 3**	850 g	25 0/7	41 DOL (urine): positive 44 DOL (blood): 5.44 × 10^5^ copies DNA/mL 44 DOL (CSF): 4.39 × 10^4^ copies DNA/mL 107 DOL (blood): 4.05 × 10^2^ copies DNA/mL	42 DOL: positive	IgG-seropositive and IgM-seronegative	Ganciclovir 6 mg/kg (39 days) + Valcyte 15 mg/kg (6 months)	persistent cholestasis, fever, elevated inflammation markers (negative blood and CSF culture), asymptomatic thrombocytopenia	good general condition, 12 months (9 months CA), specialist care—neurologist, neonatologist, ophthalmologist, medical rehabilitation
**Patient 4**	800 g	26 5/7	26 DOL (urine and blood): positive 29 DOL (blood): 1.02 × 10^4^ copies DNA/mL 43 DOL (blood): negative	19 DOL: positive	reactivity in both antibody classes (IgG 143.0 U/mL, IgM 1.22 U/mL)	Ganciclovir 6 mg/kg (6 days) + Valcyte 15 mg/kg (14 days)	pneumonia	good general condition, 7 months (4 months CA), specialist care—neurologist, neonatologist, medical rehabilitation
**Patient 5**	800 g	26 0/7	62 DOL (urine): positive 63 DOL (blood): 1.2 × 10^5^ copies DNA/mL 89 DOL (blood): negative	63 DOL: 1.82 × 10^3^ copies DNA/mL	never serologically tested for CMV infection before	Valcyte 15 mg/kg (26 days)	persistent cholestasis	good general condition, 7 months (4 months CA), specialist care—neurologist, neonatologist, ophthalmologist, medical rehabilitation

## Data Availability

Data is unavailable due to patients’ privacy and ethical restrictions.

## Abbreviations

The following abbreviations are used in this manuscript:

HCMV	Human Cytomegalovirus
cCMV	Congenital Cytomegalovirus Infection
aCMV	Acquired Cytomegalovirus Infection
NICU	Neonatal Intensive Care Unit
iNO	Inhaled Nitric Oxide
HFNC	High-Flow Nasal Cannula
DOL	Day Of Life
EEG	Electroencephalogram
CRP	C-reactive Protein
PCT	Procalcitonin
DART	Dexamethasone: A Randomized Trial
CMV-SLS	CMV-Related Sepsis-Like Syndrome
ROP	Retinopathy of Prematurity
BPD	Bronchopulmonary Dysplasia
AP-ROP	Aggressive Posterior Retinopathy of Prematurity
VEGF	Vascular Endothelial Growth Factor
VAP	Ventilator-Associated Pneumonia
PVL	Periventricular Leukomalacia
VLBW	Very Low Birth Weight
MOM	Mother’s Own Milk
NEC	Necrotizing Enterocolitis
PVD	Pulmonary Vascular Disease
MRI	Magnetic Resonance Imaging
CA	Corrected Age
